# Protocols—more structure, less ‘*Wuthering Heights*’

**DOI:** 10.1186/s13063-019-3865-7

**Published:** 2019-11-28

**Authors:** Shaun Treweek

**Affiliations:** Health Services Research Unit, University of Aberdeen, Health Sciences Building, Foresterhill, Aberdeen, AB25 2ZD UK

Many years ago I went through a phase of reading classic novels, books written by authors we’ve all heard of who are now dead. I ended up keeping just one of these: *Wuthering Heights*. Emily Brontë’s novel centres on the fiery love between man of mystery Heathcliff and Catherine Earnshaw, the latter of whom inconveniently dies halfway through the book. On Catherine’s death, Heathcliff’s desperate response is:

*And I pray one prayer—I repeat it till my tongue stiffens—Catherine Earnshaw, may you not rest as long as I am living! You said I killed you—haunt me, then!…Be with me always—take any form—drive me mad! only do not leave me in this abyss, where I cannot find you!*

Gripping stuff. The reason for raising this topic is that finding this quote in the book was hard work despite my knowing it was in there somewhere. The presentation format of *Wuthering Heights* does not make finding things easy; you’re supposed to start at the beginning and keep going.^1^ For a novel this hardly matters; few people pick up *Wuthering Heights* just to find out what Heathcliff said when Catherine died.

But it does matter for trial protocols. People do look at these protocols just to find out how the randomisation was done, or what the primary outcome is, or how adverse events will be collected. Even when information is present in a protocol, finding it can be frustratingly difficult. This is a sorry state of affairs precisely because we *do* dip in and out of protocols so much. The Standard Protocol Items: Recommendations for Interventional Trials (SPIRIT) checklist [1] recommends what should be in a protocol but not where it should be. Consequently, navigating a protocol can be maddening. Apart from wasting everyone’s time, it makes it easier for methodological weakness, error and poor practice to pass unseen.

In 2015 our late founding editor Doug Altman suggested that we should make greater use of structure in research articles [2]. Structured information of the sort seen in trial registries is in contrast to what I think of as the ‘*Wuthering Heights*’ approach to research reporting: material written so that you have to start at the beginning and keep going. But we are not writing novels. We are providing information that should be clear, complete and, crucially, easy to navigate.

To make protocols less ‘*Wuthering Heights*’, *Trials* is experimenting with a new way to structure the protocol for a randomised trial. The simple innovation is to include all the SPIRIT headings and item identifiers in the protocol itself. We then know what has to be in the protocol and where to put it. We can see—quickly—when information is missing. If you, the authors, want to add additional headings, specific to your trial, go ahead. If you’d like to add the pdf of the protocol you submitted to your ethics committee (in any language) as a supplementary document, please do.

We’ve reformatted the protocol for the AMBER trial, which was published in *Trials* in 2017 [3], into this new structured format so that you can compare it to the original; see Additional file 1. A newly submitted protocol has also used this format [4] (https://trialsjournal.biomedcentral.com/articles/10.1186/s13063-019-3500-7). In both cases you’ll see that it’s not so different to what we’re all used to. These protocols do, however, have something written under all 51 items of the SPIRIT checklist, which was not quite the case for the AMBER protocol that had been published already. To make things easier, we’ve created a template for this new format that contains both SPIRIT and *Trials* guidance in one place: https://trialsjournal.biomedcentral.com/submission-guidelines/preparing-your-manuscript/study-protocol/structured-study-protocol-template. We’ve changed the ordering of some SPIRIT items in the template to make it flow better, but all the original SPIRIT item identifiers are there. If what matters to you is how harms are collected and assessed, searching for ‘{22}’ in one of these structured protocols will always land you in the right place. Typing ‘{6b}’ will always take you to the rationale behind the comparators. In short, readers can search the headings, find what is needed and ignore the rest if they want to.

*Trials* is not making use of this structure mandatory, and we will continue to consider protocols submitted in other formats. All will continue to be checked against the SPIRIT checklist. But we think the new structured approach will improve reporting (as it did for the previously published AMBER protocol) and improve publication times because the cross-check with SPIRIT will be much easier. The protocol is likely to be a more useful tool for those inside and outside the trial team who need to refer to it. As Doug wrote in 2015, despite improved communication about trials being part of the editorial that launched *Trials*, there hasn’t been much activity on this theme [2]. Well, here’s to structured protocols.

## Supplementary information

**Additional file 1.** The AMBER protocol after reformatting using the structured protocol template.

## Data Availability

Not applicable.

## Footnotes

1 Readers interested in a 4-min musical abstract of *Wuthering Heights* should check out the 1978 song of the same name by British icon Kate Bush: https://www.youtube.com/watch?v=-1pMMIe4hb4.
