# Supplementary material for: Protocols—more structure, less ‘Wuthering Heights’
Source: Trials. 2019 Nov 28;20:649. doi: 10.1186/s13063-019-3865-7 (PMC6883621; doi:10.1186/s13063-019-3865-7)
Supplement: Supplementary file 1 — Additional file 1. The AMBER protocol after reformatting using the structured protocol template. [file 13063_2019_3865_MOESM1_ESM.pdf]

This protocol was published in Trials on 29th March 2017: https://doi.org/10.1186/s13063-017-1890-y

The text is exactly as published except for text marked in pink, which was added while reformatting the protocol to the new SPIRIT-based template developed by Trials. It shows that the new template has helped to improve reporting in a few places. Otherwise changes are to formatting only.

Title

Abdominal massage for neurogenic bowel dysfunction in people with multiple sclerosis (AMBER — Abdominal Massage for Bowel Dysfunction Effectiveness Research): study protocol for a randomised controlled trial

Names of protocol contributors

McClurg D[1], Goodman K[1], Hagen S[1], Harris F[2], Treweek S[3], Emmanuel A[4,5], Norton C[6], Coggrave M[6], Doran S[1], Norrie J[3], Donnan PT[7], Mason H[8] and Manouklan S[8].

Abstract

Background: Multiple sclerosis (MS) is a life-long condition primarily affecting younger adults. Neurogenic bowel dysfunction (NBD) occurs in 50–80% of these patients and is the term used to describe constipation and faecal incontinence, which often co-exist. Data from a pilot study suggested feasibility of using abdominal massage for the relief of constipation, but the effectiveness remains uncertain.

Methods/design: This is a multi-centred patient randomised superiority trial comparing an experimental strategy of once daily abdominal massage for 6 weeks against a control strategy of no massage in people with MS who have stated that their constipation is bothersome. The primary outcome is the Neurogenic Bowel Dysfunction Score at 24 weeks. Both groups will receive optimised advice plus the MS Society booklet on bowel management in MS, and will continue to receive usual care.

Participants and their clinicians will not be blinded to the allocated intervention. Outcome measures are primarily self-reported and submitted anonymously. Central trial staff who will manage and analyse the trial data will be unaware of participant allocations. Analysis will follow intention-to-treat principles.

Discussion: This pragmatic randomised controlled trial will demonstrate if abdominal massage is an effective, cost-effective and viable addition to the treatment of NBD in people with MS.

Keywords: Multiple sclerosis, Constipation, Abdominal massage, Randomised controlled trial

Administrative information

Note: the numbers in curly brackets in this protocol refer to SPIRIT checklist item numbers. The order of the items has been modified to group similar items (see http://www.equator-network.org/reporting-guidelines/spirit-2013-statement-defining-standard-protocol-items-for-clinical-trials/).

Title {1}	Abdominal massage for neurogenic bowel dysfunction in people with multiple sclerosis (AMBER — Abdominal Massage for Bowel Dysfunction Effectiveness Research): study protocol for a randomised controlled trial
Trial registration {2a and 2b}	ClinicalTrials.gov, ISRCTN85007023. Registered on 10 June 2014
Protocol version {3}	Version 5; 02.02.17
Funding {4}	National Institute for Health Research Health Technology Assessment programme, project number HTA 12/127
Author details {5a}	[1]NMAHP RU, Glasgow Caledonian University, A603 Govan Mbeki Building, Glasgow, G4 0BA, UK. [2]NMAHP RU, Stirling University, Stirling, UK. [3]Health Services Research Unit, University of Aberdeen, Aberdeen, UK. [4]University College Hospital, University College London, London, UK. [5]National Hospital for Neurology and Neurosurgery, University College London, London, UK. [6]King's College London, London University College, London, UK. [7]Tayside Clinical Trials Unit, University of Dundee, Dundee, UK. [8]Yunus Centre for Social Business and Health, Glasgow Caledonian University, Glasgow, UK.

Name and contact information for the trial sponsor {5b}	Yasmin Glover, RIE, Glasgow Caledonian University, Cowcaddens Road, Glasgow, G4 0BA, UK
Role of sponsor {5c}	The sponsor played no part in study design; collection, management, analysis, and interpretation of data; writing of the report; and the decision to submit the report for publication.

Introduction

Background and rationale {6a}

Multiple sclerosis (MS) is a life-long condition primarily affecting younger adults. There is currently no accurate data on the exact number of people with MS in the UK, but it is thought to be between 107,000 and 127,000 [1] and is growing by around 2.4% per year. Bowel problems occur much more often in people with MS than in the normal population. Neurogenic bowel dysfunction (NBD) is the broad term used to describe constipation and faecal incontinence (FI) secondary to neurological disease or trauma and is caused by damage to the nerves controlling colonic function. In addition confounding factors such as side effects of medication, poor diet and decreased exercise may compound symptoms. Constipation in people with MS is usually due to slow colonic transit time and can be exacerbated by pelvic floor dyssynergia and may lead to the individual becoming housebound and spending long periods of time trying to empty their bowels, thus limiting their ability to work [2]. In severe cases impaction occurs, which often requires hospital admission. FI may co-exist or exist independently, and it is often described as the most devastating event imaginable, leading to social and emotional issues and devastating psychosocial consequence [2].

The costs arising from treating bowel and bladder problems in people with MS totalled £11 M for National Health Service (NHS) England in 2013/14 [3, 4]. People with MS are twice as likely to have a non-elective admission for constipation as members of the general population with an average admission cost of £1729 [3, 4].

Bowel management often plays a significant part in the lives of people with MS, but there is little robust evidence on effective interventions [2]. Initial management of NBD includes conservative measures such as modification of diet and fluids, laxatives or constipating medication, rectal interventions such as digital rectal stimulation and manual evacuation of stool, suppositories/enemas progressing to more invasive and expensive interventions such as rectal irrigation and surgery (e.g. stoma). Current evidence for the effectiveness of abdominal massage, the focus of this trial, has been summarised in a Cochrane Review under- taken by the Chief Investigator [5]. The review found 11 randomised controlled trials (RCTs) with a total of 268 participants. One of these studies was a pilot trial of people with MS and abdominal massage, which confirmed feasibility and was used to determine the sample size of this RCT [6]. Overall the review authors concluded that although the results were promising, there was a lack of unequivocal evidence of effectiveness for abdominal massage, and they recommended further trials be carried out in specific populations [5]. As to the mechanism of action, there was even less information, with only a small study in a spinal cord injury (SCI) population suggesting that changes in anorectal physiology parameters could be detected during abdominal massage and recommending further exploration [7, 8].

This background research has led us to design a robust pragmatic trial to determine the effectiveness and cost- effectiveness of abdominal massage in people with MS who have bothersome constipation. The corresponding flow chart for the Abdominal Massage for Bowel Dysfunction Effectiveness Research (AMBER) trial is shown in Fig. 1, and the schedule of enrolment, interventions and assessments is provided in Fig. 2.

It is hypothesized that the effectiveness and cost-effectiveness of a strategy of abdominal massage with optimised advice is superior to optimised advice without abdominal massage in terms of clinical and cost- effectiveness at 24 weeks.

Objectives {7}

The AMBER trial will determine/undertake the following:

1. Is an optimised bowel care programme with abdominal massage more effective and cost-effective compared to an optimised bowel care programme alone in reducing the

symptoms of NBD in people with MS?

2. To identify and investigate via a process evaluation the possible mediating factors that have an impact on the effectiveness of the intervention (including intervention fidelity), how these mediating factors influence effectiveness, and whether the factors differ between the randomised groups.
3. To undertake a formal economic evaluation of the interventions from an NHS and societal perspective with a focus on the NHS and the participants.
4. The physiological effect of abdominal massage on the bowel (substudy at one of the sites).
5. To validate responsiveness of a new NBD quality of life questionnaire.

Trial design {8}

The present study is a multi-centred, parallel group, superiority, RCT comparing the effectiveness of an experimental strategy of once daily abdominal massage for 6 weeks against a control strategy of no massage in people with MS who have stated that their constipation is bothersome. Randomisation is at a 1:1 ratio for the two arms of the trial.

An integral part of the project will be the process evaluation which will follow a mixed methods, longitudinal, case study design [9, 10]. In addition at one site only, anorectal physiology studies to determine mechanism will be conducted in consenting patients in both groups.

Methods: Participants, interventions and outcomes

Study setting {9}

Routine care setting for MS patients in the UK.

Eligibility criteria {10}

Participants will be included on the condition that they are over 18 years old (both male and female), have a diagnosis of MS (in a stable phase, i.e. no MS relapse for 3 months), have had no major change in medication for 1 month, have had no abdominal massage for at least 2 months and are bothered by their constipation.

Exclusion criteria are inability to undertake the massage themselves or the lack of a carer willing to do it; those who are unable to understand the study or give informed consent; or those who have contraindications to massage, e.g. history of abdominal/pelvic cancer, hiatus, inguinal or umbilical hernia, rectal prolapse, inflammatory bowel disease, pregnancy, past history of volvulus, indwelling urinary catheters, recent abdominal scars, abdominal wounds or skin disorders that may make abdominal massage uncomfortable. Recent sudden and severe changes in bowel habits and rectal bleeding are not exclusion criteria but will be flagged and discussed with the local Principal Investigator before recruiting to the study.

Who will take informed consent? {26a}

All participants will undergo a process of informed consent which will include the delivery of balanced written information concerning the need and overall benefit of the trial followed up by discussion with a local AMBER researcher. This discussion will include a check of understanding concerning benefits and risks of participation and ensuring that participants accept that the treatment will be allocated at random regardless of any personal preference they may have.

The consent form indicates if the participant would like to be contacted to discuss possible interviews (for the process evaluation). If the participant indicates yes, the process evaluation researcher gets in touch with the participant and if they agree to take part, a subsequent consent form is signed.

Additional consent provisions for collection and use of participant data and biological specimens {26b}

The consent form asks if participants would be happy to be contacted in the future about research participation. Participants can say no to this and still part in the AMBER trial.

Interventions

Explanation for the choice of comparators {6b}

The comparator is optimised bowel care. During the outpatient appointment the participants' existing routine bowel care will be reviewed and optimised. For example, dietary and fluid advice will be given as well as encouragement to be more active and use a better defaecation position. This advice is recommended standard care. For the duration of the trial, we will ensure that this happens, something that may not always be the case in routine health care.

Intervention description {11a}

Both trial arms

Both intervention and control groups will receive a 6-week intervention consisting of one outpatient consultation followed by weekly telephone calls to review adherence. Thus both groups will have the same amount of health professional contact. Both groups will receive what we have termed 'optimised bowel care'. Often, in people with MS, bowel care is delivered in a haphazard fashion with little standardisation or guidelines for treatment. Optimised care (delivered to both groups) will include advice which will be reinforced by providing the Bowel Care Advice Leaflet of the MS Society.
The intervention and control groups will differ as described:

1. Control group (optimised bowel care). During the outpatient appointment the participants' existing routine bowel care will be reviewed and optimised. For example, dietary and fluid advice will be given as well as encouragement to be more active and use a better defaecation position.
2. Intervention group (abdominal massage and optimised bowel care). In addition to optimised bowel care as described for the control group, the intervention researcher, who will have been trained in the massage, will teach the participant and/or their carer how to deliver the abdominal massage. This will include viewing a short study-specific DVD showing the massage being given to a patient and self-massage techniques, a study-specific abdominal massage training booklet as well as a demonstration of the technique on the participant. During the training the carer or participant will try the various strokes and will be able to ask questions. Possible adaptations to accommodate a participant's disability will also be discussed. It is recommended that

the abdominal massage be included as part of the participant's usual bowel care regimen. Daily application was recommended in our pilot study in people with MS, and this was found to be acceptable with 80% adherence reported in the massage diary [6].

Both groups will have weekly telephone calls (lasting approximately 10 minutes; weeks 1–6) where they will discuss any changes/difficulties with bowel management. The intervention group participants will also discuss frequency of use and any problems in using abdominal massage.

Participants randomised to the control group will be given access to the training materials for the massage at the end of their follow-up visit (24 weeks). Each trial centre will decide if they will hold practical training sessions for their control participants and will make them aware of this at the baseline visit.

Abdominal massage technique

The ideal position of the participant is supine, with appropriate head and knee support, in a relaxed atmosphere. Adaptations to this position may be required depending on the patient's disability. The training videos provide demonstrations for both supine and sitting and self- and carer-led massage.

There are four basic strokes with the massage lasting about 10 minutes.

1. Stroking commences from the small of the back and follows the dermatome of the vagus nerve, over the iliac crests and down both sides of the pelvis towards the groin.
2. Effleurage follows the direction of the ascending colon across the transverse colon and down the descending colon. This is also repeated several times with increasing pressure.
3. Palmar kneading tracks down the descending colon, up the ascending colon and down the descending colon once again. Effleurage is repeated and continued with a relaxing transverse stroke over the abdomen.
4. Vibration over the abdominal wall to relieve flatus concludes the massage session.

Criteria for discontinuing or modifying allocated interventions {11b}

There are no special criteria for discontinuing or modifying allocated interventions. Participants may choose to stop doing the abdominal massage themselves for any reason. Those in the comparison group will receive standard care.

Strategies to improve adherence to interventions {11c}

None beyond normal encouragement.

Relevant concomitant care permitted or prohibited during the trial {11d}

No special provisions.

Provisions for post-trial care {30}

None beyond standard care within the National Health Service.

Outcomes {12}

Primary outcome measure

The primary outcome is the difference between the intervention and the control group in the change in the Neurogenic Bowel Dysfunction Score (NBDS) [10] at 24 weeks. The NBDS is a 10-item questionnaire covering frequency of bowel movements, headache, perspiration or discomfort during defaecation; medication for constipa- tion or faecal incontinence; time spent on defaecation; frequency of digital stimulation or evacuation; frequency of faecal incontinence; flatus; and perianal skin problems.

The maximum score is 47; a score over 14 is considered severe [11].

Secondary outcome measures

Bowel outcomes are assessed with the following measures:

- Constipation symptoms. The Constipation Scoring System (CSS) [12] is used. It measures constipation symptoms via an 8-item questionnaire with items on frequency of bowel movement, difficulty with evacuation, feeling of incomplete evacuation, pain, length of time for evacuation, assistance with evacuation, number of failed attempts and the duration of constipation. Maximum score is 30, with higher scores indicating

greater severity.

- Bowel symptoms. A 7-day bowel diary is used to record the frequency of bowel movement, time spent defaecating, stool type (Bristol stool chart) [13], laxative use, additional interventions, e.g. digital stimulation, changes in medication and contact with NHS staff. The diary will be completed for 7 days on three occasions: prior to trial entry, during weeks 1–6 and at week 23.

Adherence to massage schedule

A massage diary will be used. The massage intervention will be recorded in the diary during the intervention period (1–6 weeks) for the intervention group and will be used as part of compliance monitoring.

Urinary outcomes

Bladder function will be measured using the Qualiveen Questionnaire Short Form [14], which consists of an 8- item questionnaire assessing bladder dysfunction, such as leakage and signs of incomplete voiding. Often if patients with MS are suffering from constipation, they report that their bladder symptoms are worse, especially urgency and frequency, which can lead to an increase in urinary incontinence. By using such an outcome measure the effect of the change in bowel function on the bladder can be assessed.

Quality of life outcomes

The following quality of life assessments will be used:

- For health status the EuroQol five-dimension questionnaire (EQ-5DL) [15] generic instrument will be used. Trial participants will complete the EQ-5DL at baseline, at 6 weeks and at 24 weeks post randomisation. This instrument will provide quality of life weights to compute quality-adjusted life years (QALYs).
- The Neurogenic Bowel Impact Score (NBIS, a patient-reported symptom and quality of life questionnaire for NBD) will also be used. One investigator (MC) is developing a patient-reported symptom and quality of life questionnaire for NBD as part of a National Institute for Health Research (NIHR)-funded postdoctoral fellowship. This questionnaire will be completed at all time points to allow assessment of the measure's

sensitivity to change. The development of such a questionnaire is important for research and clinical practice in this area.

Economic outcomes

The cost and use of NHS services will be collected via a ‘Patient resource questionnaire’. The cost to the patients and their families/carers will also be collected via the Patient resource questionnaire). From this information we will calculate the incremental costs, QALYs and incremental cost per QALY.

Radio-opaque marker transit tests and anorectal physiology tests

Standard anorectal physiology tests and colonic transit studies are routinely undertaken before treatment at one site (which will recruit 30 MS participants), and the out- comes will be recorded, as they may have some predictive value. These participants will have a repeat transit study test at 24 weeks. Transit studies have been used in previous trials and have been shown to be sensitive to change [16]. Radio-opaque marker transit studies most commonly involve the ingestion of a number of Sitzmarks capsules, each containing different shaped radio-opaque markers, followed by a single plain abdominal X-ray 5 days later. These transit studies enable an assessment of total colonic transit time, but not segmental transit [17, 18]. The radio-opaque markers will be posted to the partici- pant, who will ingest them and then attend for abdominal X-ray 5 days later. Out-of-pocket expenses will be paid to the participant for attending the follow-up transit study. This is an internal pilot study of the feasibility of under- taking such tests within this population and their compliance with attending for the repeat tests.

Process evaluation

Meso-level and micro-level contextual data on the intervention sites will be gathered in order to explore pre- existing background contexts and any changes (e.g. in local capacity) that might have an impact on delivery or take-up of the intervention. These data will be gathered primarily by undertaking interviews with relevant parties as detailed in Table 1.

Table 1 Process evaluation data collection

Details
Documentary analysis related to health care trusts; local capacity/ budgets at 10 implementation sites
Semi-structured interviews ($n = 85$)
• 20 patients in intervention arm (interviewed twice)
• 2 staff members from each site ($n = 10$ sites), interviewed twice
• 5 stakeholder interviews
Bowel diary analysis (5 patients)

Participant timeline {13}

See Fig 1.

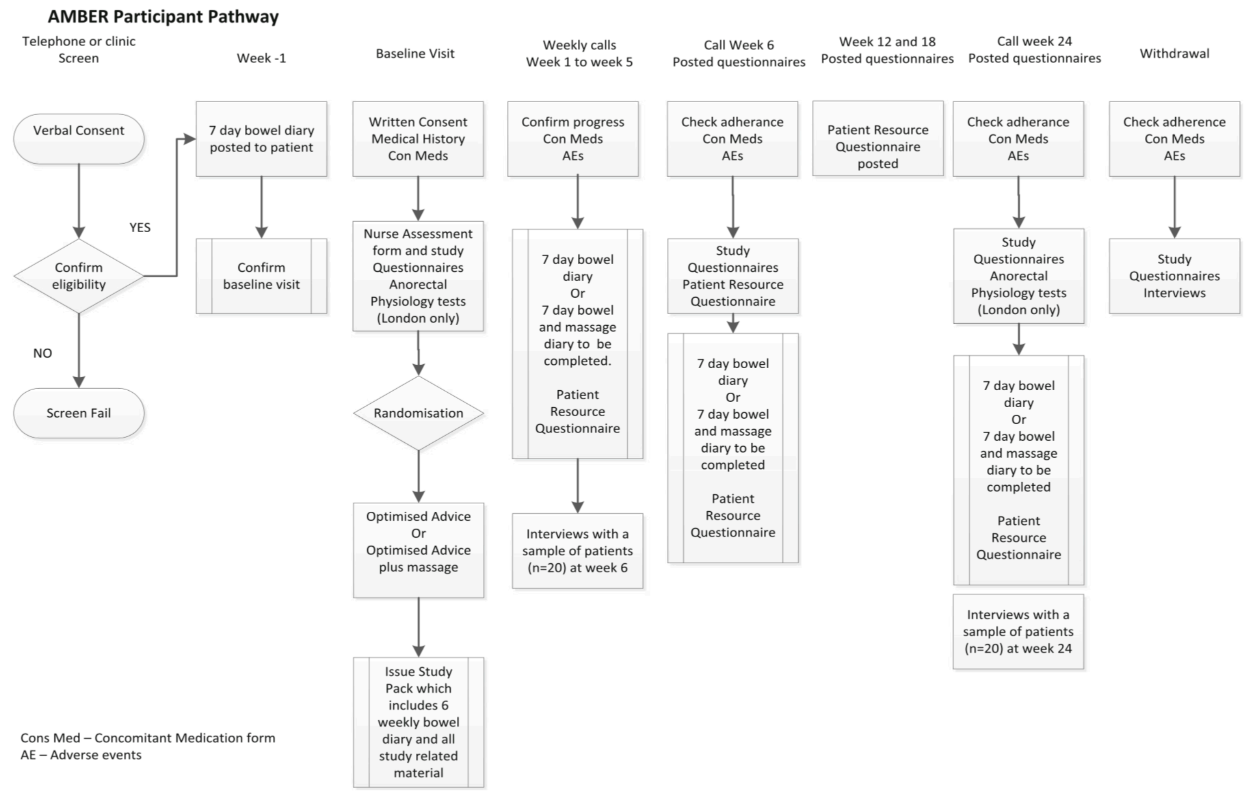

Fig. 1 AMBER participant pathway

Sample size {14}

The only published data available on the NBDS to inform sample size calculations is from our own pilot trial in the MS population [6]. Based on these data a sample size of 60 per group was calculated as necessary to detect a difference between groups of 4.21 (standard deviation, SD 7.02) at a 5% level of significance with 90% power. Thus for a fully powered study the sample size, allowing for a 20% drop-out, is 150. However, it was suggested by the funder that this figure be reviewed and increased. Therefore we have increased our sample size to 200 (100 per group), which would allow for greater than expected attrition.

Recruitment {15}

The research team at each study centre will be responsible for identifying potential patients. Following identification a letter of introduction and an 'Expression of Interest Form' will be posted or given to patients in the routine clinic. A member of the research team will telephone those patients who express an interest to provide further information and to assess eligibility.

If eligible and willing to take part, a time for the participant to attend clinic will be arranged. An appointment letter will be posted to the participant along with a 7-day bowel diary. The participant will be asked to complete the bowel diary during the week prior to this appointment and will also be asked to bring someone who is willing to do the massage to this appointment if required.

Assignment of interventions: allocation

Sequence generation {16a}

Patients who provide written informed consent will be randomly allocated to either the optimised bowel care programme (control group) or the optimised bowel care programme

and massage (intervention group). Randomisation will be stratified by site and minimised on level of disability (walking unaided, walking aided or wheelchair bound).

Concealment mechanism {16b}

Randomisation will utilise a web-based randomisation system.

Implementation {16c}

Implementation will be by staff at the Tayside Clinical Trials Unit.

Assignment of interventions: Blinding

Who will be blinded {17a}

Due to the nature of the intervention, participants and the research team delivering the intervention will not be blinded to the treatment received. Outcome measures are primarily self-reported and submitted anonymously. Those involved in the data analyses and statistics will be blinded to the group allocation.

Procedure for unblinding if needed {17b}

Participants and clinical staff are not blinded.

Data collection and management

Plans for assessment and collection of outcomes {18a}

Data will be collected via participant-completed questionnaires at baseline and at 6 and 24 weeks. A 7-day bowel diary will be completed prior to baseline, and a bowel diary (or bowel and massage diary if allocated to intervention) during weeks 1–6 and at 24 weeks. Completed outcome data are retuned by post. Demographic and medical history information will be collected at baseline.

Patient resource questionnaires will also be sent to the participant at home to complete at 12 and 18 weeks.

Anorectal physiology and colonic transit time data will be collected from one site only where it is routinely con- ducted as part of the assessment, and these will be repeated at week 24. Figure 1 shows the AMBER participant pathway and data collected at each time point. The schedule of enrolment, interventions and assessments is provided in Fig. 2.

Plans to promote participant retention and complete follow-up {18b}

For the intervention group, nothing beyond normal encouragement. Participants randomised to the control group will be given access to the training materials for the massage at the end of their follow-up visit (24 weeks), which may serve as an incentive to remain involved in the trial.

	STUDY PERIOD								
	Enrolment	Allocation	Post-allocation						Close-out
TIMEPOINT	$-t_1$ Week-1	t_0 week 0	t_1 week 1	t_2 week 2	t_3 week 3	t_4 week 4	T_5 week 5	T_6 week6	t_x week 24
ENROLMENT:									
Eligibility screen and verbal consent	X								
Informed consent		X							
Allocation		Randomised							
INTERVENTIONS:									
[Intervention A] Abdominal massage + advice		Shown massage + Advice	Abdominal massage daily + advice<br>Weekly Follow-up support calls by nurse						
[Intervention B] Control group =Advice		Advice	Advice<br>Weekly Follow-up support calls by nurse						
ASSESSMENTS:									
Bowel Diary or Bowel and massage diary	x		x	x	x	x	x	x	x
Primary outcome (Neurogenic Bowel dysfunction score)		x						x	x
Qol Questionnaires		x					.	x	x
Patient resource Questionnaire			x	x	x	x	x	x	x
Con med use		x	x	x	x	x	x	x	x
Adverse Events			x	x	x	x	x	x	x

Fig. 2 Schedule of enrolment, interventions, and assessments for 'Abdominal massage for neurogenic bowel dysfunction in people with multiple sclerosis'

Data management {19}

All trial participants are given an individual trial number which will be used on all Case Report Forms for that participant. Data will be entered into the secure trial database by the data coordinator based at the AMBER central office at Glasgow Caledonian University.

Confidentiality {27}

All collected information will be kept strictly confidential and will be stored in accordance with the UK Data Protection Act 1998 and retained in accordance with the latest Directive on Good Clinical Practice (GCP) and local policy.

Plans for collection, laboratory evaluation and storage of biological specimens for genetic or molecular analysis in this trial/future use {33}

None.

Analysis

Statistical methods for primary and secondary outcomes {20a}

Analysis will be performed for the intention-to-treat population and reported in accordance with the Consolidated Standards of Reporting Trials (CONSORT) statement [19, 20] and the International Conference on Harmonisation (ICH) E9 'Statistical Principles in Clinical Trials' [21]. All trial data will be summarized by treatment group and total. Continuous data will be reported as mean (SD), categorical data as N (%). Primary outcome analysis will be a general linear model comparing the difference in mean NBDS score at 24 weeks between the intervention and control groups with adjustment for the minimisation covariates and baseline score. Other covariates will be considered for further adjustment and if necessary stated in the statistical analysis plan prior to data lock. Secondary analysis will use similar analysis of covariance (ANCOVA) models, correcting for baseline characteristics. A two-sided p value of 0.05 will be taken as significant for each outcome.

Interim analyses {21b}

There will be no interim analyses.

Methods for additional analyses (e.g. subgroup analyses) {20b}

Subgroup analyses

Subgroup analyses will be carried out by first testing for a subgroup factor by trial group interaction. If this is significant at the 5% level, results will be estimated separately by the different subgroups. These analyses will also be repeated for all the secondary outcomes. This will include a secondary analysis comparing those who undertook the massage themselves to those receiving carer-delivered massage. Appropriate transformations of outcomes will be per- formed where necessary to satisfy modelling assumptions.

Process evaluation analysis

Interviews will be coded and analysed using techniques of framework analysis [22], assisted by QSR NVivo 10. The analysis will pay particular attention to barriers and facilitators to uptake of abdominal massage for MS pa-ients, synthesising staff and patient views into an overall implementation narrative. Staff interviews will be analysed longitudinally, using a framework matrix to explore developments over the two time periods.

Progress tracking data for trial recruitment and adherence will be synthesised in narrative form with themes from the qualitative data; thus the mixed methods will complement each other rather than be used to triangulate and verify either data source. The process evaluation will also draw on the results of the participant massage diaries, seeking to explain adherence/intensity rates via qualitative interview data. Informed by the realist evaluation approach, the analysis will seek to identify key mechanisms involved in the implementation of the intervention, barriers and facilitators to success and what might impinge on outcomes. Finally, the lessons learned from the process evaluation will provide analytical input into the optimisation of the intervention for future implementation into practice if effectiveness is demonstrated.

Economic analysis

The cost of abdominal massage and optimal bowel care relative to optimised bowel care alone in people with MS who have NBD will be considered from NHS and societal perspectives. Health care resource use by patients in both trial arms will be collected at each of the follow-up time periods (i.e. weeks 1–6, 12, 18 and 24). This will include contact with health professionals and medications prescribed. These will be costed using NHS pay and prices or, where appropriate, using other (e.g. market-based) sources. Health-related quality of life will be assessed using the EQ-5DL questionnaire completed by all patients at baseline, at 6 weeks and at 24 week follow-up. QALYs associated with each arm of the trial will be calculated using the UK tariff scores (utility values) from the EQ-5DL descriptive system [23, 24].

Data on intervention costs and patient resource use will be aggregated and the statistical significance of differences in cost per patient between trial arms assessed by appropriate methods depending on the distributional characteristics of the data. Depending on the outcome measure, which would be QALYs, NBDS or both, if there is no statistically significant evidence that one treatment strategy is more effective than another, a cost-minimisation framework will be used, and the less expensive form of care will be recommended. If one strategy appears to be dominant (i.e. to be more effective and less costly than the alternative), the uptake will be recommended. If one form of care appears to be more effective and more expensive than the comparator, estimates of incremental cost-effectiveness (and cost-utility) ratios will be generated.

Methods in analysis to handle protocol non-adherence and any statistical methods to handle missing data {20c}

The analysis will be intention-to-treat with no account taken of protocol non-adherence.

The extent of missing data will be explored in the outcomes, especially the primary outcome. Patterns of missing data will be explored and predictors of missingness examined, especially if these vary by intervention. If necessary, multiple imputations will be utilised to impute missing data assuming the missingness mechanism is missing at

random (MAR). A detailed statistical analysis plan will be agreed to before the end of data entry and before the treatment code is broken.

Plans to give access to the full protocol, participant level-data and statistical code {31c}

This document is the full protocol. Anyone interested in other data or documentation should contact the corresponding author.

Oversight and monitoring

Composition of the coordinating centre and trial steering committee {5d}

The trial will be overseen by a Project Management Group (PMG), a Trial Steering Committee (TSC). The PMG will have a teleconference approximately every 4–6 weeks during the recruitment period and then bimonthly after this. The group will support any decision-making that the trial management team need further advice on. The TSC will have an independent chairperson and members but also includes the trial collaborators. Four TSC meetings are planned.

Composition of the data monitoring committee, its role and reporting structure {21a}

The Data Monitoring Committee will oversee trial safety and consider trial progress, especially recruitment and retention status. It will have an independent chair (a statistician) and report to the Trial Steering Committee. Three meetings are planned. All statistical reports to the DMEC will be prepared by a statistician from the Trials Unit.

Adverse event reporting and harms {22}

The AMBER trial involves treatments which are well established in clinical practice for individuals with MS who have bothersome constipation; therefore adverse events (AEs) (although these are unlikely) will be those observed in everyday practice associated with

optimised bowel care and abdominal massage. Expected AEs arising from the treatments are noted below and thus will not be collected as AEs but noted in the weekly follow-up data collection.

- Increased flatulence
- Abdominal cramps
- Stomach rumblings/noises
- Loose stool, which in some instances may lead to faecal incontinence.

All AEs and serious AEs will be assessed for serious- ness, causality, severity and expectedness and will be reported to the relevant regulatory bodies.

Frequency and plans for auditing trial conduct {23}

The Trial Steering Committee and Data Monitoring Committee will meet every six months.

Plans for communicating important protocol amendments to relevant parties (e.g. trial participants, ethical committees) {25}

Funders, sponsors and National Health Service Research & Development Offices will be notified routinely and appropriate approvals gained and communicated as required by them and by the trial sponsor.

Dissemination plans {31a}

In addition to journal publications and conference presentations, we will develop a publication and dissemination policy and will discuss presentations and dissemination with relevant patient and clinical interest groups.

Discussion

This study is a pragmatic patient-oriented trial aiming to capture a true representation of the actual patient population of interest. We know from previous work and from reviews that there is a lack of evidence-based interventions for NBD accompanied by a lack of willingness in patients and indeed clinicians to discuss such intimate problems. As discussed earlier the cost to the NHS and to the patient is considerable and increasing; moreover the effect on quality of life both for patients and carers is significant and disabling. The lack of robust evidence on effective management leads to inconsistent advice and confused management pathways. MS is a long-term condition, and supported self-management is important. Abdominal massage as an adjunct to treatment offers a safe, non-invasive and non-drug intervention which could be undertaken by the patient or a carer. As such it is likely to be an attractive option for many.

Should the trial demonstrate that abdominal massage is effective we will have the necessary information from our process evaluation to identify the barriers and enablers to implementation of such a self-management technique. Easy integration into standard local pathways should be possible, as the training required for clinicians and patients (or carers) is minimal. The process evaluation will also identify if this training has been adequate.

Trial status

The AMBER trial is currently recruiting in 12 UK centres and is recruiting to target. The first patient was randomised on 22 January 2015, and recruitment is due to end June 2016 and follow-up completed by the end of December 2016. The trial has both a TSC and a DMEC, and both oversight groups have convened twice. The DMEC reviewed their report in December 2015 and had no issues with the trial. They commended the trial team on recruiting to target. For the trial protocol and updates see Clinical-Trials.gov and the trial website http://www.gcu.ac.uk/amber/. The trial registration number is ISRCTN85007023.

Abbreviations

AE: Adverse events; Cons Med: Concomitant medication; CSS: Constipation Scoring System; CTU: Clinical Trials Unit; DMEC: Data Monitoring and Ethics Committee; HTA: Health technology assessment; ISRCTN: International Standard Randomised Controlled Trial Number; MS: Multiple sclerosis; NBD: Neurogenic bowel dysfunction; NBDS: Neurogenic Bowel Dysfunction Score; NETSCC: NIHR Evaluation, Trials and Studies Coordinating Centre; PMG: Project Management Group; QALY: Quality-adjusted life year; REC: Research Ethics Committee; SAE: Serious adverse event; SOP: Standing operating procedure; TCTU: Tayside Clinical Trials Unit; TSC: Trial Steering Committee

Acknowledgements

We would like to acknowledge Kerry Walker as our database manager and would like to thank all local Principal Investigators and site staff for their commitment in recruitment for the AMBER trial. We would like to acknowledge Tayside Clinical Trials Unit (CTU) for their invaluable support of the study.

Authors' contributions {31b}

All named authors adhere to the authorship guidelines of *Trials*. All authors have agreed to publication.

DM is the lead grant holder and Chief Investigator, conceived the study, led on the protocol development, contributed to writing the manuscript and approved the final version for publication. FH and SD led on process evaluation aspects of the protocol development and contributed to writing the manuscript and approved the final version for publication. KG contributed to the trial management and to writing the manuscript and approved the final version for publication. PD led on statistical protocol development and approved the final manuscript for publication. SM and HM led on health economic aspects of protocol development and approved the final manuscript for publication. SH, ST, AE, CN, MC, PD and JN are co-investigators on the grant, contributed to study design, clinical expertise and protocol development and approved the final manuscript for publication.

All named authors adhere to the authorship guidelines of *Trials*. All authors have agreed to publication.

Funding {4}

The trial was funded by the United Kingdom National Health Service through the National Institute for Health Research Health Technology Assessment programme, open call project number HTA 12/127.

The views and opinions expressed therein are those of the authors and do not necessarily reflect those of the Health Technology Assessment Programme, NIHR, NHS or the Department of Health. The authors also acknowledge the support of the NIHR through the Comprehensive Clinical Research Network.

Availability of data and materials {29}

Any data required to support the protocol will be supplied on request.

Ethics approval and consent to participate {24}

The trial has ethical approval from West of Scotland Research Ethics Committee 4, obtained on 11 June 2014 (14/WS/0111), and central and local NHS site recruitment approvals have all been obtained. The study is sponsored by Glasgow Caledonian University, and the AMBER trial office is based in the Nursing, Midwifery and Allied Health Professions Research Unit (NMAHP-RU).

Participants have the right to withdraw from the study at any time and for any reason, and all participants are made aware that withdrawal will not affect their routine care.

Consent for publication {32}

Consent for publication is not applicable as there are no identifying images of other personal details of participants presented. See supplementary materials for informed consent forms.

Competing interests {28}

The authors declare that they have no competing interests.

Author details

[1]NMAHP RU, Glasgow Caledonian University, A603 Govan Mbeki Building, Glasgow, G4 0BA, UK. [2]NMAHP RU, Stirling University, Stirling, UK. [3]Health Services Research Unit, University of Aberdeen, Aberdeen, UK. [4]University College Hospital, University College London, London, UK. [5]National Hospital for Neurology and Neurosurgery, University College London, London, UK. [6]King's College London, London University College, London, UK. [7]Tayside Clinical Trials Unit, University of Dundee, Dundee, UK. [8]Yunus Centre for Social Business and Health, Glasgow Caledonian University, Glasgow, UK.

References

1. Mackenzie IS, et al. Incidence and prevalence of multiple sclerosis in the UK 1990-2010: a descriptive study in the General Practice Research Database. J Neurol Neurosurg Psychiatry. 2014;85(1):76–84.
2. Coggrave M, Wiesel PH, Norton C. Management of faecal incontinence and constipation in adults with central neurological diseases. Cochrane Database Syst Rev. 2006. Issue 2. Art. No. CD002115. doi: 10.1002/14651858.CD002115.pub3.
3. Thc Cost of Constipation Report Coloplast 1https://www.coloplast.co.uk/ Global/UK.../ Cost_of_Constipation_Report_FINAL.PDF. Accessed 20 May 2015.
4. Thomas S, Mynors G, Simpson S, Meade N, Bowen A. Measuring the burden of hospitalisation in Multiple Sclerosis: A cross-sectional analysis of the English Hospital Episode Statistics database 2009-2014. London: NHiS Commissioning Excellence and Multiple Sclerosis Trust; November 2015. Publication number: 0001.
5. McClurg D, Hagen S, Dickinson L. Abdominal massage for the treatment of constipation. Cochrane Database Syst Rev. 2011 Issue 4. Art. No.: CD009089. doi: 10.1002/14651858.CD009089.
6. McClurg D, Hagen S, Hawkins S, Lowe-Strong A. Abdominal massage for the relief of constipation in people with multiple sclerosis. Mult Scler. 2011;17(2):223–33.
7. Coggrave M, Norton C, Wilson-Barnett J. Management of neurogenic bowel dysfunction in the community after spinal cord injury: a postal survey in the United Kingdom. Spinal Cord. 2009;47(4): 323–30. doi:10.1038/sc.2008.137.
8. Glickman S, Kamm MA. Bowel dysfunction in spinal cord injury patients. Lancet. 1996;347(9016): 1651–3.

9. Craig N, Dieppe P, Macintyre S, Michie S, Nazareth I, Petticrew M. Developing and evaluating complex interventions: new guidance. London: MRC; 2008.
10. Anderson R. New MRC guidance on evaluating complex interventions: clarifying what interventions work by researching how and why they are effective. BMJ. 2008;337(a1937):doi: 10.1136/bmj.a1937.
11. Krogh K, Christensen P, Sabore S, Laurberg A. Neurogenic bowel dysfunction score. Spinal Cord. 2006;44(10):625–31.
12. Agachan F, Chen T, Pfiefer J, et al. A constipation scoring system to simplify evaluation and management of constipated patients. Dis Colon Rectum. 1996;39(6):681–5.
13. Lewis SJ, Heaton KW. Stool form scale as a useful guide to intestinal transit time. Scand J Gastroenterol. 1997;32(9):920–4. doi:10.3109/00365529709011203. PMID9299672.
14. Bonniaud V, Bryant D, Parratte B, Guyatt G. Development and validation of the Short Form of a Urinary Quality of Life Questionnaire: SF-Qualiveen.
J Urol. 2008;180(6):2592–8.
15. Nowels D, McGloin J, Westfall JM, Holcomb S. Validation of the EQ-5D quality of life instrument in patients with myocardial infarction. Qual Life Res. 2005;14(1):95–105.
16. Rao SSC, Ozturk R, Laine L. Clinical utility of diagnostic tests for constipation in adults: a systematic review. Am J Gastroenterol. 2005;100:1605–15.
17. Cowlam S, Vinayagam R, Khan U, Marsden S, Minty I, Moncur P, Bain I, Yiannakou YJ. Blinded comparison of faecal loading on plain radiography versus radio-opaque marker transit studies in the assessment of constipation. Clin Radiol. 2008;63:1326–31.
18. Lundin E, Graf W, Garske U, Nilsson S, Maripuu E, Karlbom U. Segmental colonic transit studies: comparison on a radiological and a scintigraphic method. Colorectal Dis. 2006;9:344–51.
19. Schulz KF, Altman DG, Moher D, for the CONSORT Group. CONSORT 2010 Statement: updated guidelines for reporting parallel group randomised trials. BMC Med. 2010;8:18.
20. Moher D, Hopewell S, Schulz KF, Montori V, Gøtzsche PC, Devereaux PJ, Elbourne D, Egger M, Altman DG. CONSORT 2010 explanation and elaboration: updated guidelines for reporting parallel group randomised trials. J Clin Epidemiol. 2010;63(8):e1–37.
21. 21. International Conference on Harmonisation of Technical Requirements for Human Use. ICH harmonised tripartite guideline statistical principles for clinical trials E9. 1998. www.ich.org/products/guidelines/efficacy.html. Accessed June 2016.
22. 22. Richie J, Spencer L. Qualitative data analysis for applied policy research. In: Bryman A, Burgess B, editors. Analysing qualitative data. London: Routledge; 1994. p. 173–94.
23. 23. Devlin N, Shah K, Feng Y, Mulhern B, van Hout B. Valuing health related quality of life: an EQ-5D-5L value set for England. OHE Research Paper. London: Office of Health Economics; 2016.
24. 24. Feng Y, Devlin N, Shah K, Mulhern B, van Hout B. New methods for modelling EQ-5D-5L value sets: an application to English data. OHE Research Paper. London: Office of Health Economics; 2016.
